# SERUM CONCENTRATIONS OF LOSARTAN METABOLITES CORRELATE WITH IMPROVED PHYSICAL FUNCTION IN A PILOT STUDY OF PREFRAIL OLDER ADULTS

**DOI:** 10.1093/geroni/igad104.0067

**Published:** 2023-12-21

**Authors:** Peter M Abadir

**Affiliations:** Johns Hopkins University, Baltimore, Maryland, United States

## Abstract

DOI: 10.1093/gerona/glac102

